# The impact of COVID-19 on the achievement of public school students in British Columbia: A multilevel analysis on the results from the province-wide standardized assessment

**DOI:** 10.1016/j.heliyon.2025.e42851

**Published:** 2025-02-19

**Authors:** Fubiao Zhen

**Affiliations:** Department of Educational Psychology, Texas A&M University, 540 Ross St Suite 704, College Station, TX, 77843, USA

## Abstract

This study aims to investigate the impact of COVID-19 on public school students' achievement by collecting and analyzing the two-year test results from the Foundational Skills Assessment, a province-wide standardized test administered annually in British Columbia, Canada. Multilevel modeling was conducted to analyze whether the impact of COVID-19 correlated with local school district demographics, including the percentage of indigenous students, the percentage of students with special needs, and the median household income. The results revealed that the impact of COVID-19 on students’ literacy and numeracy achievement was associated with the percentage of students with special needs and median household incomes in the local school districts. Implications of the findings for postpandemic revitalization were discussed.

## Highlights

• No strong evidence of negative COVID-19 impact on general students' achievement.
• Schools with more indigenous students achieved lower across tests and grades.
• The achievement of students with special needs were more sensitive to school closures.
• Students from lower-income households were more vulnerable to learning losses.

## 1 Introduction

There is a worldwide concern about the impact of COVID-19 on students' academic achievement. Researchers have pointed out that the unprecedented school closure caused by the pandemic has had a strong influence on how teachers instruct their students [[1], [2], [3]] and put pressure on parents to educate their children at home [[4], [5], [6]]. Standardized test results from different countries and regions confirm how the drastic change in educational conditions and the time spent at home has affected students' learning outcomes. For instance, based on the US National Center of Educational Statistics [7], in 2022, public school students achieved an average of three points lower in fourth-grade and eighth-grade reading than in 2019; and an average of five points lower in fourth-grade math and eight points lower in the eight-grade math than in 2019. Similarly, according to the *National Dataset* report of the UK [8], the percentage of students in year three working at or above age-related expectations in autumn 2021 dropped 14 % in reading, 21 % in writing, and 14 % in math compared with the percentages in autumn 2019. The report further pointed out that the negative impact of COVID-19 on students’ standardized test results was strong and long-lasting. It is shown that younger students and students from underrepresented groups were more vulnerable to the impact. As such, recent researchers have advocated for educators and educational policy-makers to immediately address the negative impact of lost time in-class school learning [[9], [10], [11]]. These researchers also argued that schools need long-term efforts to track students back to the prepandemic learning trajectory.

However, in the province of British Columbia (BC), Canada, the official provincial report of the Ministry of Education [12] claimed that the province of BC “may have experienced fewer impacts than other jurisdictions” (p.5). This speculation was based on the comparatively lower COVID-19 prevalence and less time lost to school closure in BC than other jurisdictions in Canada [12]. Compared with reports from other regions [7,8], the relatively less severe impact of COVID-19 on education reported by the government of BC left a critical aspect in the discussion. Given that the COVID-19 prevalence and the corresponding policies on school closure vary from different countries or regions, it would be invalid to assume that the impact of COVID-19 is similar across jurisdictions. Furthermore, in the BC provincial report, the analyses of COVID-19 impact focused mainly on completion rates of 12th grade graduates and school absenteeism. It also highlighted the urgent need for education officials and policy-makers to have analyses of standardized test results to better understand the different impacts of COVID-19.

Therefore, this study addressed this issue by conducting in-depth analyses of the results of the Foundational Skills Assessment (FSA), which is the province-wide standardized test of BC administrated annually at grades four and seven. Considering that BC is one of the regions with comparatively lower COVID-19 prevalence and a shorter period of school closure, I think this study could provide a more comprehensive understanding of the impact of COVID-19 on students’ academic achievement. Furthermore, district-level characteristics including the percentage of underrepresented students (i.e., indigenous students and students with special needs) and local district median household income were also collected and analyzed to explore the interactive effects of district demographics and conditions during the pandemic.

## 2 Impact of COVID-19 on education outcomes as measured by standardized assessments

Starting in March 2020, most countries implemented school closures as a major response to the spread of the COVID-19 pandemic [13]. Under that condition, education researchers expressed concerns about the interruptions school closures could cause in students' learning and development [3,14,15]. As Sievertsen and Burgess [15] pointed out, missing school days and peer interactions could decelerate students' academic skill growth. It is stressed further that home education would unlikely fully replace the instructions students received at school [6,15]. In the early stage of the investigation, researchers projected the long-term impact of school closures caused by COVID-19 on students' educational development by analyzing previous data and studies about absenteeism and school vacations in the US [16,17]. By collecting data from more than five million of students’ learning loss after summer vacations and synthesizing previous studies about absenteeism, Kuhfeld et al. [16] projected that US students would experience learning loss in reading and math across grades during the school closure caused by COVID-19. In addition, it is also noted that students from low-socioeconomic status (SES) districts would be especially vulnerable to learning loss [16,17].

Follow-up studies [18,19] analyzing standardized test scores during the COVID-19 pandemic reflected initial projections by Kuhfeld et al. [16]. By applying the Measures of Academic Progress (MAP) assessment, a computer-based assessment developed by Northwest Evaluation Association (NWEA), Kuhfeld et al. [18] analyzed the test scores of 5.4 million students in grades three to eight in the US and tracked the two-year changes in reading and math scores from fall 2019 to fall 2021. Based on their findings, grade three to eight students scored relatively lower in math and reading in fall 2021 in comparison with their peers in fall 2019. The achievement gap between low-poverty and high-poverty elementary schools widened by an extra 0.1 to 0.2 standard deviations. In another study conducted by Halloran et al. [20], the research team collected and analyzed the standardized assessment results and demographic information from 12 US states. The research findings indicated that the pass rates of English language arts and math tests decreased in the school year 2020–2021. Halloran et al. [20] deemed the school year 2020–2021 as “the pandemic year” during which lower pass rates in English language arts and math tests were related to fewer days of in-person instruction. Furthermore, by combining school district-level demographic information, it was also found that school districts with a higher percentage of Black, Hispanic students or students who were eligible for free and reduced-price lunches, were more vulnerable to the test score decrease in English language arts. However, although underrepresented student groups were emphasized by researchers [18,20] as more vulnerable to the learning loss caused by school closure, research studies focused specifically on indigenous students and students with special needs are still very limited.

## 3 Impact of COVID-19 on indigenous students

Following the definition of Aboriginal People in Section 35 of the Constitution Act, the indigenous people in the province of BC were defined as first nations (status or nonstatus), Inuit, and Métis peoples. According to statistics Canada [21], there were more than 1.8 million indigenous people in Canada, which was 5 % of the total population. The province of British Columbia (BC) had the second largest population of indigenous people in this country. In 2021, there were 290,210 indigenous citizens living in the province of BC, accounting 16.34 % of the total indigenous population in Canada. In contrast to the large population of indigenous people, the educational inequity indigenous students have been experiencing is not fully addressed by the BC educational system. The achievement gap between indigenous students and nonindigenous students has been prevailing in BC. According to the BC Ministry of Education [22], the public-school completion rate of indigenous students for the school year 2021–2022 was 72 %, which was 18 % lower than the BC residents’ average.

Researchers have long been investigating the educational inequity of indigenous students and exploring the factors behind the inequity [[23], [24], [25]]. Harper and Thompson [26] argued that indigenous students confronted “structural oppression” (p.1) in Canadian education, as indigenous students had limited access to high-quality education, health care, and food. Furthermore, the Canadian education system failed to remove the feeling of inferiority that indigenous students may experience [26,27]. Such structural problems were exacerbated by the COVID-19. During the pandemic, due to the lack of educational funding [25,28], indigenous students were likely to be further marginalized in school education. It was found that indigenous students, in compared with their nonindigenous peers, had less access to the infrastructure and technological support for high-quality online learning [29,30], which was essential for education quality and continuity during school closures. According to the *COVID-19 Student Impacts* report [12], indigenous students were among the lowest in SES in the BC public school system. During the pandemic, indigenous students were more likely to transit to homeschooling and less likely to return or graduate from secondary education than their nonindigenous peers. In December 2020, the absenteeism rates of indigenous students reached more than 20 %, which was 10 % higher than the student average. Unfortunately, analyses of indigenous students’ learning outcomes (as measured by standardized tests during the COVID-19 pandemic) has not been fully examined by educators or researchers. Our understanding of how the COVID-19 pandemic impacted (and continues to impact) the learning outcomes of indigenous students and whether the impact widened the achievement gap between indigenous and nonindigenous students, remains vague.

## 4 Impact of COVID-19 on students with special needs

Similar to indigenous students, the students with special needs also facing extra obstacles and difficulties during the pandemic [31,32]. According to the BC Ministry of Education [33], students with special needs were defined as those “who have a disability of an intellectual, physical, sensory, emotional or behavioral nature, have a learning disability or have special gifts or talents” (p.1). The Ministry of Education [33] requires that all school boards in BC provide appropriate special education programs to ensure that students with special needs have “equitable access to learning, achievement, and the pursuit of excellence” (p. 5). However, researchers [34,35] found that instruction for students with special needs was inequitably interrupted due to school closures caused by the pandemic. During school closures, students with disabilities confront the loss of educational support and medical supplies [34,36]. In a case study, MacDonald and Hill [35] conducted in-depth interviews with six parents with school-aged children and nine school teachers in BC. The researchers focused on documenting the experience of parents and teachers during the COVID-19 pandemic and their attitudes toward the rapid changes in instructional formats from in-person to online. Based on the investigation, MacDonald and Hill [35] found that students with disabilities were especially vulnerable to the suspension of in-class instruction, as it was difficult for schools to coordinate learning assistance for those with special needs during the school closures. It was also found that online instructions could not fully address their learning needs. Similar findings were also reported by the analysis results of the Spring 2020 American Educator Panels (AEP) COVID-19 Survey [37,38], in which 1000 teachers in the American public schools across grades from K to 12 were collected and analyzed. It was found that more than half of the teachers (58 %) indicated in the survey that they did not received adequate support for students with severe disabilities. Approximately 30 percent teachers did not received adequate support for students with mild or moderate disabilities [38].

Furthermore, as most public schools switched to online learning during the pandemic, differentiated instructions provided by schools to gifted and talented students were highly restricted [39]. Rogers [40] and Kitsantas et al. [41] argued that support for gifted and talented students was a continuous challenge even during the regular school years. Enrichment needs to be provided in gifted student programs to ensure educational success for those students [41]. However, during the school closure, gifted and talented students were further blocked from enrichment programs in schools, or interactions with their gifted peers. For instance, Wolfgang and Snyderman [39] collected and analyzed the thoughts and perceptions of 110 parents and 53 gifted-student support teachers. Based on participant responses to open-ended interview questions, the researchers found that gifted services were directly impacted by COVID-19. Gifted students were less likely to be enriched during the school closures, and the lack of interactions with other gifted peers could cause social-emotional issues and intensify anxiety.

## 5 Household income and educational attainment during the COVID-19 pandemic

Despite COVID-19, researchers have widely documented the correlation between household income and educational attainment [[42], [43], [44], [45], [46]]. Studies found that lower household income means limited physical or psychological resources for students' academic development [46,47]. Low household income could restrict children's brain development which is highly associated with school readiness skills [48,49]. According to previous study findings, parents from low-income families spend less time in their children's education [50,51] or less frequently engage in communications or interactions with their children [52,53]. During the COVID-19 pandemic, the academic development of children from low-SES families was intensively impacted by the pandemic in many different countries or regions [7,8,18]. Researchers [54,55] have pointed out that children living in low-income families were less likely to have high-quality education during school closures, as children from low-income families had limited resources for homeschooling. During the pandemic, the implementation of online instructions required digital devices and internet connections, and successful homeschooling required a comfortable and stable home environment, grade-level appropriate books or learning materials, and supports from parents [56]. For instance, Andrew et al. [57] collected survey responses from 5582 parents in England. Based on their analysis, it was found that students from low-income families had fewer educational supplies and support and spent significantly less time studying during school closures. Additionally, the school closure restricted access to healthy food and health care provided by schools, which were crucial for the educational development of children from low-income families. Therefore, the achievement gap between children from low-income and high-income families was likely to widen during school closures.

In summary, research studies and standardized assessment results have widely documented the negative consequences of COVID-19 on students’ learning outcomes. The impact of COVID-19 on some of the underrepresented student groups, such as ethnic minority students or students from low-SES households, could be more intensive. However, among the studies investigating the impact of COVID-19 on vulnerable populations, the evidence based on standardized assessment results analyses was still limited. The analysis of standardized assessment results could be constructive in comprehensively understanding the impact of COVID-19 on education. Another critical issue overlooked in the previous research is how the impact of COVID-19 (and government response) varies in different countries or regions. It would be invalid to investigate the impact of COVID-19 on education in a region without considering the local conditions. Therefore, in this study, the issue was addressed by delimiting this study to the impact of COVID-19 measured by the Foundation Skills Assessment (FSA), a province-wide standardized assessment in BC. By analyzing the FSA results in a province with a relatively short period of school closure and a lower prevalence of COVID-19, this study aimed to provide more information about how the impact of COVID-19 was related to the local conditions of school districts. Specifically, BC public school students reading, writing, and numeracy achievement measured by FSA were analyzed and whether the district factors, such as the percentage of indigenous students, percentage of students with special needs, and the local-district median-household income had any impact on the FSA achievement during the pandemic was examined. Three primary and secondary research questions are addressed in the study:

Primary Research Question.

1. Is there any impact of COVID-19 on fourth and seventh grade public school students' Foundation Skills Assessment achievement?

Secondary Research Questions.

2. Does the percentage of indigenous students or students with special needs in any given district have any interactive effect with COVID-19 on fourth- and seventh-grade Foundation Skills Assessment achievement at the district level?
3. Does the median household income have any interactive effect with COVID-19 on fourth- and seventh-grade Foundation Skills Assessment achievement at the district level?

## 6 Method

In response to the advice of the Provincial Health Officer, the Government of British Columbia (BC), suspended all in-class instruction on March 17, 2020, because of the COVID-19 pandemic. By June 2020, in-class instruction resumed at 50 % capacity, and by September 2020, it was fully resumed at 100 % capacity. This made BC one of the two jurisdictions in Canada with the shortest period of in-class instruction suspension [12].

### 6.1 Data source

The data used in this study were obtained from the open-access data pool on the Government of BC website [58]. The average FSA achievement scores of all districts in BC (*N* = 60), as well as the average percentage of indigenous students and students with special needs in each district in the school years 2019–2020 and 2020–2021 were collected. In addition, the median household income in each district for the years 2020 and 2021 of each district provided by the Government of BC [58] was also collected. Detailed demographic information on school districts in BC is exhibited in Table 1.

**Table 1:** Demographic information of school districts in BC.

	Grade 4	Grade 7
Number of districts	60	60
Percentage of indigenous students (%)		
Average	24.94	24.05
*SD*	19.97	19.05
Min.	1.94	1.65
Max.	100	100
Percentage of students with special needs (%)		
Average	11.91	17.57
*SD*	4.10	5.52
Min.	0	10.66
Max.	22.79	35.48
Median household income (in thousands)		
Mean	88.10	
*SD*	12.87	
Min.	64.43	
Max.	118.72	

Among the 60 school districts in BC, a large variation in the percentage of indigenous students and students with special needs was observed. Form 2019–2020 to 2020–2021, the average percentage of indigenous students was 24.94 in grade four and 24.05 in grade seven, with some of the school districts serving 100 percent of indigenous students in the two years. The average percentage of students with special needs was 11.91 in grade four and 17.57 in grade seven. For median household income in the two years, the lowest district had 64.43 thousand Canadian dollars and the highest district had 118.72 thousand Canadian dollars.

### 6.2 Foundation Skills Assessment

The Foundation Skills Assessment (FSA) is a province-wide, standardized assessment of student academic knowledge in BC. The BC Ministry of Education administers this assessment annually for all BC students in grades four and seven, including students with disabilities and diverse abilities. Schools are required to provide proper accommodations to students with special needs. The assessment included three components: tests of reading, writing, and numeracy. It is generally administered in the fall semester each year from October to November, except during the 2020–2021 school year, where the FSA was administered in the spring semester from January 18th to February 26th for extra preparation time following the school closure.

According to the BC Ministry of Education [59], the skill assessment of reading and writing was based on the provincial English Language Arts Curriculum. This curriculum was used by the ministry to form a basis to assess students’ “ability to critically analyze and make meaning from diverse texts and to communicate and express oneself in a variety of modes and for a variety of purposes in relevant contexts” (p.6). Based on the mathematics curriculum, the skills assessment of numeracy measures “the ability to interpret information within a given situation, apply mathematical understanding to solve an identified problem and analyze and communicate a solution” (p.6). The skills assessment contains two types of question formats: the selected-response format, including multiple-choice questions, matching, and sequencing questions, and the constructed-response format, including short/extended answer questions, problem-solving questions, and writing assignments. The Ministry of Education [59] introduced the Foundation Skills Assessment (FSA) as a technically sound instrument designed and developed by following an evidence-centered design approach [60].

### 6.3 Data analysis

To comprehensively understand the impact of COVID-19 on Foundation Skills Assessment (FSA) results, a series of research analyses were performed. First, a descriptive analysis of FSA results in the school years 2019–2020 and 2020–2021 was conducted. Next, considering that the FSA results were collected consecutively over two years in the same set of school districts (*N* = 60) and that the main focus of the research study was to investigate how the impact of COVID-19 was related to the district-level factors (i.e., percentage of indigenous students, percentage of students with special needs, median household income), the hierarchical linear modeling (HLM) technique [61] was used in this study. In this multilevel model, the time level (coded as 0 for the 2019–2020 school year and 1 for the 2020–2021 school year) was seen as the first-level data nested within school districts. Therefore, the time level was set as level one, and the district level was set as level two. As each district was measured two times, so there were totally 120 results in each FSA test in the multilevel model. The HLM version-seven software was used in the analysis.

#### 6.3.1 Model 1

First, an unconditional model was formulated without adding any covariates. In this model, the intra-class correlation (ICC) was estimated for each test. The model was specified as follows:

Level-1 of model-1: ${\text{FSA}}_{ij}=\beta_{0j}+r_{ij}$

Level-2 of model-1: $\beta_{0j}=\gamma_{00}+u_{0j}$In this model,

${\text{FSA}}_{ij}$ represents FSA achievement for year *i* in district *j*.

$\beta_{0j}$ represents the average FSA achievement of district *j*.

$\gamma_{00}$ represents the grand mean across all assessments.

$r_{ij}$ represents the random error associated with level-1, *Var* ($r_{ij}$) = $\sigma^{2}$.

$u_{0j}$ represents the random error associated with level-2, *Var* (${\;u}_{0j}$) = $\tau_{00}$.

The ICC for each assessment to measure the proportion of between-group variance [62] was calculated by using the equation:

(1) $$\rho =\frac{\tau_{00}}{\tau_{00}+\sigma^{2}\;}$$

#### 6.3.2 Model 2

In the second model, the year of FSA administration was added as the level-1 variable. The level-1 of the model is specified as:

(2) $${\text{FSA}}_{ij}=\beta_{0j}+\beta_{1j\;}\left({\text{Year}}_{\text{ij}}\right)+r_{ij}$$

$\beta_{0j}$ represents the mean achievement of district *j*.

$\beta_{1j}$ represents the effect of time, and

$r_{ij}$ represents the random error associated with this level.

#### 6.3.3 Model 3

The third model is the full model where the district level factors were added based on model 2 to investigate the effects of district-level characters on FSA achievements. The level-2 of the model is depicted as:

$$\beta_{0j}=\gamma_{00}+\gamma_{01}\;\left({\text{Indegious}}_{j}\right)+\gamma_{02}\;\left({\text{Special}\;\text{needs}}_{j}\right)+\gamma_{03}{\;\left({\text{Household}\;\text{income}}_{j}\right)+u}_{0j.}$$

$$\beta_{1j}=\gamma_{10}+\gamma_{11}{\;\left({\text{Indegious}}_{j}\right)+\gamma }_{12}\;\left({\text{Special}\;\text{needs}}_{j}\right)+\gamma_{13}\;\left({\text{Household}\;\text{income}}_{j}\right)$$

In this level, $\gamma_{00}$ is the grand mean of FSA overall assessments. The percentages of indigenous students, percentages of students with special needs, and the median household income of the districts were added as variables to control for possible differences in FSA achievement ($\gamma_{01}$, $\gamma_{02}$, $\gamma_{03}$, respectively) and possible moderate effects on time ($\gamma_{11}$, $\gamma_{12}$, $\gamma_{13}$, respectively). The random error at this level is represented by $u_{0j}$. Therefore, the full model combining the levels 1 and 2 was:

(3) $${\text{FSA}}_{ij}=\gamma_{00}+\gamma_{10}\;\left({\text{Year}}_{ij}\right)+\gamma_{01}\;\left({\text{Indegious}}_{j}\right)+\gamma_{02}\;\left({\text{Special}\;\text{needs}}_{j}\right)+\gamma_{03}{\;\left({\text{Household}\;\text{income}}_{j}\right)+\gamma }_{11\;}\left({\text{Year}}_{ij}\times {\text{Indegious}}_{j}\right)+\gamma_{12}\;\left({\text{Year}}_{ij}\times {\text{Special}\;\text{needs}}_{j}\right)+\gamma_{13\;}\left({\text{Year}}_{ij}\times {\text{Household}\;\text{income}}_{j}\right)+u_{0j}+r_{ij}$$

## 7 Results

Based on the descriptive analysis, it was found that only the FSA writing average scores dropped in the school year 2020–2021, which was the year with school closures. For the fourth grade, the average score of FSA writing was 0.01 lower in the 2020–2021 school year (*M* = 2.08, *SD* = 0.26) than in the 2019–2020 school year (*M* = 2.09, *SD* = 0.33). For the seventh grade, the average score of FSA writing was 0.03 lower in the 2020-2021school year (*M* = 2.22, *SD* = 0.26) than in the 2019–2020 school year (*M* = 2.19, *SD* = 0.32). No strong evidence of the COVID-19 impact was reflected in the descriptive analysis of FSA results. However, the number of participants revealed an unexpected direction. In the 2020–2021 school year, the number of public-school students who participated in FSA dropped by more than 3000 (approximately 10–15 percent less of the previous year's total) in each test compared to the number from the last year. The number of participants in the seventh grade FSA reading test had the most significant decrease in the 2020–2021 school year: BC public schools had 4241 fewer students participating in the reading test than in the last year (Table 2).

**Table 2:** Descriptive statistics of FSA results.

Grade	FSA	2019–2020			2020–2021		
		Number of students	Mean	*SD*	Number of students	Mean	*SD*
4	Reading	29,613	469.21	32.11	26,555	486.63	30.07
	Writing	28,298	2.09	0.33	25,230	2.08	0.26
	Numeracy	29,528	468.33	39.44	26,417	481.12	39.46
7	Reading	30,538	456.74	32.98	26,297	459.04	38.18
	Writing	28,817	2.22	0.26	24,616	2.19	0.32
	Numeracy	30,418	453.28	45.21	26,199	464.59	42.42

Note. In grades 4 and 7 FSA, the best possible score is 800 for Reading and Numeracy, and 12 for Writing.

In the multilevel analysis, the ICC (equation (1)) for each assessment were calculated (Table 3). For the fourth grade, the ICCs for FSA reading, writing, and numeracy tests were 0.74, 0.78, and 0.84 respectively, indicating that 74 % of the variance in FSA reading, 78 % of the variance in FSA writing, and 84 % of the variance in FSA numeracy were between group (i.e., school district) variance. For the seventh grade, the ICC is 0.94 for FSA reading, 0.75 for FSA writing, and 0.90 for FSA numeracy. Therefore, 94 % of the variance in FSA reading, 75 % of the variance in FSA writing, and 90 % of the variance in FSA numeracy were between group variance.

**Table 3:** Unconditional model for FSA results in grades four and seven.

Grade 4						
Parameter	Reading		Writing		Numeracy	
	Coeff.	*S.E.*	Coeff.	*S.E.*	Coeff.	*S.E.*
Fixed effects						
Intercept ($\gamma_{00}$)	**477.25**∗∗	3.93	**2.08∗∗**	0.04	**474.22∗∗**	4.94
Random effects						
School level ($u_{0j}$)	**784.51∗∗**	174.18	**0.07∗∗**	0.02	**1338.86∗∗**	269.53
Residual ($r_{ij}$)	**276.57∗∗**	52.05	**0.02∗∗**	0.00	**248.70∗∗**	46.28

Grade 7						
Parameter	Reading		Writing		Numeracy	
	Coeff.	*S.E.*	Coeff.	*S.E.*	Coeff.	*S.E.*
Fixed effects						
Intercept ($\gamma_{00}$)	**456.80∗∗**	4.71	**2.20∗∗**	0.04	**458.10∗∗**	5.56
Random effects						
School level ($u_{0j}$)	**1282.89∗∗**	244.22	**0.06∗∗**	0.01	**1751.89∗∗**	340.57
Residual ($r_{ij}$)	**88.14∗∗**	16.47	**0.02∗∗**	0.00	**204.76∗∗**	38.11

Note: ∗*p* < .05; ∗∗*p* < .01; Coeff. = Model Coefficient.

Based on the results of model two (equation (2)), no significant negative effect of time ($\gamma_{10}$) was found on either of the tests. In contrast, as reflected by the descriptive analysis results, both fourth and seventh grade students improved their FSA reading and numeracy scores significantly in the 2020–2021 school year. The results indicated that, in general, school closure had no significantly negative effect on students’ FSA test results, or the extra preparation time remediated the negative effect on FSA assessment results successfully (Table 4).

**Table 4:** Hierarchical linear model for FSA results with year of administration as the predictor in grades four and seven.

Grade 4						
Parameter	Reading		Writing		Numeracy	
	Coeff.	*S.E.*	Coeff.	*S.E.*	Coeff.	*S.E.*
Fixed effects						
Intercept ($\gamma_{00}$)	**469.21∗∗**	4.06	2.09	0.04	**468.33∗∗**	5.07
Year ($\gamma_{10}$)	**16.41∗∗**	2.21	−0.01	0.03	**12.13∗∗**	2.46
Random effects						
School level ($u_{0j}$)	**848.48∗∗**	170.13	**0.07∗∗**	0.02	**1369.28∗∗**	267.47
Residual ($r_{ij}$)	**141.53∗∗**	26.47	**0.02∗∗**	0.00	**175.87∗∗**	32.7

Grade 7						
Parameter	Reading		Writing		Numeracy	
	Coeff.	*S.E.*	Coeff.	*S.E.*	Coeff.	*S.E.*
Fixed effects						
ntercept ($\gamma_{00}$)	**454.39∗∗**	4.80	**2.21∗∗**	0.04	**451.07∗∗**	5.70
Year ($\gamma_{10}$)	**4.65∗∗**	1.62	−0.02	0.03	**13.52∗∗**	1.96
Random effects						
School level ($u_{0j}$)	**1301.51∗∗**	246.36	**0.06∗∗**	0.01	**1831.85∗∗**	345.75
Residual ($r_{ij}$)	**76.55∗∗**	14.29	**0.02∗∗**	0.00	**111.26∗∗**	20.70

Note: ∗*p* < .05; ∗∗*p* < .01; Coeff. = Model Coefficient.

By including school district information in model three (equation (3)), significantly negative effects of the percentage of indigenous students ($\gamma_{01}$) were found across all tests and grade levels. The results revealed that the more indigenous students a school district served, the lower their average scores would be. However, no interactive effect of time and the percentage of indigenous students ($\gamma_{11}$) was found. This indicated that although schools with more indigenous students scored averagely lower across FSA tests, there was no substantial evidence to support the assumption that the achievement gap between indigenous and non-indigenous students in FSA was widened by school closures (Table 5).

**Table 5:** Hierarchical linear model for FSA results with year of administration and school-level predictors in grades four and seven.

Grade 4						
Parameter	Reading		Writing		Numeracy	
	Coeff.	*S.E.*	Coeff.	*S.E.*	Coeff.	*S.E.*
Fixed effects						
Intercept ($\gamma_{00}$)	**497.74∗∗**	22.29	**2.06∗∗**	0.26	**499.87∗∗**	28.03
Year ($\gamma_{10}$)	−13.4	16.88	0.08	0.22	4.00	20.1
Indigenous students ($\gamma_{01}$)	**−83.04∗∗**	14.7	**−0.56∗∗**	0.17	**−121.24∗∗**	18.49
Special needs ($\gamma_{02}$)	80.07	60.53	1.91∗	0.71	134.00	76.13
Income ($\gamma_{03}$)	−0.24	0.21	0.00	0.00	−0.26	0.27
Time × Indigenous students ($\gamma_{04}$)	−14.15	11.14	−0.07	0.15	10.98	13.26
Time × Special needs ($\gamma_{05}$)	−20.24	45.85	**−2.28∗∗**	0.60	**−126.16∗**	54.57
Time × Income ($\gamma_{06}$)	**0.40∗**	0.16	0.00	0.00	0.22	0.19
Random effects						
School level ($u_{0j}$)	**195.72∗∗**	47.20	**0.025∗∗**	0.01	**322.49∗∗**	75.72
Residual ($r_{ij}$)	**76.71∗∗**	15.59	**0.01∗∗**	0.00	**111.52∗∗**	22.08

Grade 7						
Parameter	Reading		Writing		Numeracy	
	Coeff.	*S.E.*	Coeff.	*S.E.*	Coeff.	*S.E.*
Fixed effects						
Intercept ($\gamma_{00}$)	**438.13∗∗**	28.15	**1.90∗∗**	0.26	**431.19∗∗**	36.56
Year ($\gamma_{10}$)	19.64	14.92	−0.04	0.24	25.28	16.81
Indigenous students ($\gamma_{01}$)	**−78.80∗∗**	21.04	**−0.43∗**	0.2	**−158.33∗∗**	27.33
Special needs ($\gamma_{02}$)	−72.30	66.69	−0.61	0.63	62.72	86.62
Income ($\gamma_{03}$)	**0.57∗**	0.27	**0.01∗**	0.00	0.56	0.35
Time × Indigenous students ($\gamma_{11}$)	−15.07	11.15	0.24	0.18	**30.56∗**	12.57
Time × Special needs ($\gamma_{12}$)	13.90	35.35	0.34	0.57	−54.21	39.84
Time × Income ($\gamma_{13}$)	−0.16	0.14	0.00	0.00	−0.11	0.16
Random effects						
School level ($u_{0j}$)	**454.58∗∗**	94.04	**0.03∗∗**	0.01	**797.94∗∗**	161.41
Residual ($r_{ij}$)	**74.32∗∗**	14.17	**0.02∗∗**	0.00	**94.37∗∗**	18.00

Note: ∗*p* < .05; ∗∗*p* < .01; Coeff. = Model Coefficient.

In addition, it was found that the percentage of students with special needs ($\gamma_{02}$) had no significant effect on all FSA test results except for fourth-grade writing, in which there was a significant positive effect ($\gamma_{02}$ = 1.91, *p* < .05) observed. The results indicated that on average, the percentage of students with special needs had no significantly negative effect on district-level FSA scores in the year with school closure. However, significant interactive effects of time and percentage of students with special needs in fourth-grade writing ($\gamma_{12}$ = −2.28, *p* < .01) and numeracy ($\gamma_{12}$ = −126.16, *p* < .05) were found, and the parameters were all negative (Table 3). The results suggested the study assumption of the vulnerability of students with special needs during the COVID-19 pandemic, as they showed that the school districts with a higher percentage of students with special needs developed their academic achievement at a significantly lower rate.

Furthermore, it was also found that median household income ($\gamma_{03}$) had a significant main effect on seventh-grade FSA reading ($\gamma_{03}$ = 0.57, *p* < .05) and writing ($\gamma_{03}$ = 0.01, *p* < .01) and a significant interactive effect with time on fourth-grade FSA reading ($\gamma_{13}$ = 0.40, *p* < .05). The parameters were all positive (Table 5). The results showed that school districts with higher median household income increased the fourth grade FSA reading score at a significantly higher rate during the pandemic. In addition, school districts with higher median household income had significantly higher scores in the seventh grade FSA reading and writing. The results indicated that students in school districts with a higher median household income were more likely to overcome the negative impact of COVID-19 and hence achieved higher scores in FSAs.

## 8 Discussion

This study aimed to explore the impact of COVID-19 on public school students' standardized assessment results in a province with lower COVID-19 prevalence and a shorter period of school closure. By analyzing the FSA reading, writing, and numeracy results for the school years 2019–2020 and 2020–2021, no significant negative effect of school closures on grades four and seven students’ FSA achievement was found. However, after adding district-level characteristics (i.e., the percentage of indigenous students, the percentage of students with special needs, and the district median household income) into the analyses, it was found that the impact of COVID-19 was highly associated with the district-level characteristics. The FSA achievement of indigenous students, students with special needs and students in districts with lower household income were more vulnerable to the COVID-19. A discussion of our study findings follows.

Based on the results of the descriptive analysis and multilevel analyses, there is no strong evidence of negative impact from COVID-19. Generally, public school students improved most of their FSA assessment results in the 2020–2021 school year, compared with the FSA assessment results in the 2019–2020 school year. This result seems contradictory to findings from previous studies or reports, in which the negative impact of school closure caused by the COVID-19 pandemic is reflected and evidenced by standardized assessment results and research studies from different countries or regions [7,8,18,19]. However, this study's findings actually raised up an important notion in understanding the impact of COVID-19: the negative impact of the pandemic on education is highly related to local health and educational conditions. As argued by the British Columbia (BC) Ministry of Education [12], the province of BC had a comparatively shorter period of school closure (no more than six months of in-class instruction suspension) and a lower COVID-19 prevalence. The negative impact of the in-class instruction suspension appears to have been mitigated by educators and teachers after the school reopened. Additionally, the FSA assessment was postponed to the spring of 2021 by the Education Ministry of BC for extra preparation time. This could also explain the nonnegative effect results. Nevertheless, it is worth noting that some of the impacts of COVID-19 on education could not be fully measured by the standardized assessment results. As argued, the high rate of absenteeism and transfer to home education in the 2020–2021 school year also reflected the impact of COVID-19, and minority groups such as indigenous students and students with special needs were especially vulnerable to the impact [12].

Next, the significantly negative effects of the percentage of indigenous students across FSA test results reflected the achievement gap between indigenous students and their nonindigenous peers, a situation that was reported in many previous studies [23,25,29]. However, surprisingly, there was no strong evidence that the achievement gap widened with school closure. Again, the relatively shorter period of school closure of BC schools and the extra preparation time provided for FSAs in the school year 2020/2021 could be one of the reasons. Furthermore, considering that the number of indigenous students participating in the FSA in the 2020–2021 school year dropped in higher percentages than their non-indigenous peers in all three tests [12], the participation in FSA testing after the school closure was already a sign of resilience for most of the students in the 2020–2021 school year, especially for indigenous students, who usually had limited educational resources than their nonindigenous peers. Therefore, indigenous students who participated in the FSA were likely to achieve better results than those who withdrew from testing. In the postpandemic period, educators and policy-makers should pay attention to the striking achievement gap between indigenous students and non-indigenous students in FSAs. This area continues to be a site in need of more effective intervention over the long term.

The impact of COVID-19 was more salient on students with special needs. Significant interactive effects of the percentage of students with special needs and the time spent on FSA results were found on fourth-grade FSA writing and numeracy, and the parameters were all negative. The results indicated that school districts with a higher percentage of students with special needs developed their fourth-grade FSA writing and numeracy achievement at a significantly lower rate during the pandemic. The findings resonated with previous studies or reports [35,38] in emphasizing that students with special needs were especially vulnerable and sensitive to the impact of COVID-19. Even with the shorter time of school closure, learning interruptions for students with special needs were particularly detrimental. As Houtrow et al. [34] pointed out, for students with disabilities, the impact of COVID-19 is more than just a suspension of in-class instructions but also the loss of medical supplies, personal protective equipment, special support in education, and safe access to medical providers. Wolfgang and Snyderman [39] also found that gifted students were less likely to be challenged or enriched in their gifted areas during the COVID-19 pandemic. Gifted students’ opportunities to interact with other gifted peers were also highly restricted. Future studies focusing on revitalizing education in the postpandemic period should pay special attention to this segment of the student population. Children with special needs should be a focus of evaluation and intervention to address the negative consequences of COVID-19 in education and any other similar province-wide school disruptions.

Last, the school districts with a higher median household income scored significantly higher in seventh-grade FSA reading and writing. Additionally, school districts with a higher median household income improved their scores at a significantly higher rate in fourth-grade FSA reading. Echoing with previous studies [36,51,55,56], these findings revealed that the impact of COVID-19 on students' academic achievement is highly related to students’ families of origin and their SES. One explanation could be that children from low-income districts were less likely to have a high-quality virtual learning environment, which was essential for the continuity and quality of education during the school closure. In the study of Beaunoyer et al. [54], researchers found that the COVID-19 pandemic exacerbated the preexisting “digital inequalities” (p.1), which included the inequalities in digital devices, the technology access, the technological support, and the use experience. Digital inequalities could therefore intensify the inequalities in education. Similar findings were reported by Van Lancker and Parolin [55], who emphasized that a substantial number of children from low-income households in Europe have no access to the internet, appropriate learning materials, or a comfortable environment to learn at home. Another explanation could be that parents from low-income families spend less time in teaching their children [50,57]. As Kalil and Ryan [52] found, the economic strain and family stress confronted by parents from low-income households could restrain them from interacting or educating their children. Therefore, social workers, educators, community groups and so on should be continually addressing the nutrition and learning needs of children from low-SES families. Targeted instructional strategies and material supplies should be provided for children from low-SES families to decrease the differences in achievements among students in the postpandemic period.

## 9 Conclusion

The descriptive and multilevel analyses highlight the necessity of revitalizing postpandemic education in accordance with the health and educational conditions during the pandemic. Based on the study findings, the impact of COVID-19 on students' standardized assessment is highly associated with the COVID-19 prevalence and the time ranges of school closures. For the province of BC, with a comparatively shorter time of school closure, the general impact of COVID-19 on education was not strong as evidenced by the FSA assessment results. However, it is worth noting that the FSA results do not fully measure or encapsulate the impact of COVID-19. There was a considerable decrease in public school students who participated in the FSA in the 2020–2021 school year. Therefore, the revitalization of education is not just about learning outcomes but also about public school enrollment and accessibility to high-quality education. As Zhao [63] discussed, building education after the pandemic period, educators and policy-makers should avoid wasting resources by making inaccurate estimations of learning loss. Instead, teachers and educators should apply their professional judgment to support their students in accordance with the local school conditions and their students' learning needs. In this study, it was found that a proper evaluation of COVID-19's impact on education should be based on COVID-19 control and prevention in local jurisdictions, the time range of school closures, and local school district demographics.

Furthermore, this study's findings also implied that the standardized assessment results of the total student population could not fully capture the challenges and issues encountered by underserved student groups. It is necessary to pay close attention to the impact of COVID-19 on minority student groups, especially students with special needs. Our findings revealed that schools with a higher percentage of students with special needs were more sensitive to the impact of COVID-19. By looking at only the general students' assessment results, the learning needs of students with special needs are likely to be rendered invisible. The drastic and unprecedented change in the educational environment exhausted local teachers, school nurses, and school administrators. Students with special needs, compared with nonspecial needs students, rely more on additional one-on-one support from educational assistants (EA) or learning assistant teachers (LAT) [35]. Therefore, their special educational needs were unlikely to be adequately addressed during the pandemic. Communications with special needs students and their parents and caregivers should be promoted by governments and other school-related authorities for a more comprehensive understanding of their challenges. Parents and caregivers of special needs students urgently need more individualized support from EAs and LATs. Therefore, future studies could be more specific to focus on the education experiences of students with special needs. It would be informative to provide in-depth investigation about the challenges and difficulties those underrepresented or underserved students confronted during the pandemic.

Finally, this study underscored the association between the impact of COVID-19 on education and household income. Children in the districts with higher median-household income improved their FSA achievement significantly. Extra support and effective interventions should be provided for children from low-income families. As recommended by Zhao [64] and George et al. [65], school authorities need to find ways to increase access to online resources especially in times of long-term, unplanned disruption of schools. This is especially important for children from low-income families who have limited access to high-quality virtual learning devices and stable internet connections. The equipment of digital devices and reliable access to the internet is critical, not only for short-term mitigation plans but also beneficial for the long-term development of children from low-income families. Future studies could address this issue by investigating the inequalities in the access to digital devices and internet connections, as the implementation of digital devices or online learning resources has been making an increasingly significant impact on education. How to ensure the equal access to digital devices and internet connections among students with different SES become a critical discussion about equity in education that should be addressed.

## Ethics declaration

Review and/or approval by an ethics committee was not needed for this study because the data used in this study was collected from a publicly available dataset, and no personal identifiable information was included.

## Data availability statement

The data associated with the study is deposited into a publicly available repository, and the data is openly available in the official website of the Government of British Columbia at https://www2.gov.bc.ca/gov/content/home.

## Declaration of competing interest

The authors declare that they have no known competing financial interests or personal relationships that could have appeared to influence the work reported in this paper.
